# The Evidence of Glioblastoma Heterogeneity

**DOI:** 10.1038/srep07979

**Published:** 2015-01-27

**Authors:** Akio Soeda, Akira Hara, Takahiro Kunisada, Shin-ichi Yoshimura, Toru Iwama, Deric M. Park

**Affiliations:** 1 Department of Neurosurgery, Gifu University School of Medicine, Gifu City, Gifu, Japan; 2 Department of Tumor Pathology, Gifu University School of Medicine, Gifu City, Gifu, Japan; 3 Department of Tissue and Organ Regeneration, Gifu University School of Medicine, Gifu City, Gifu, Japan; 4 Department of Neurological Surgery, University of Virginia, Charlottesville, VA, USA

## Abstract

Cancers are composed of heterogeneous combinations of cells that exhibit distinct phenotypic characteristics and proliferative potentials. Because most cancers have a clonal origin, cancer stem cells (CSCs) must generate phenotypically diverse progenies including mature CSCs that can self-renew indefinitely and differentiated cancer cells that possess limited proliferative potential. However, no convincing evidence exists to suggest that only single CSCs are representative of patients' tumors. To investigate the CSCs' diversity, we established 4 subclones from a glioblastoma patient. These subclones were subsequently propagated and analyzed. The morphology, the self-renewal and proliferative capacities of the subclones differed. Fluorescence-activated cell sorting and cDNA-microarray analyses revealed that each subclone was composed of distinct populations of cells. Moreover, the sensitivities of the subclones to an inhibitor of epidermal growth factor receptor were dissimilar. In a mouse model featuring xenografts of the subclones, the progression and invasion of tumors and animal survival were also different. Here, we present clear evidence that a brain tumor contains heterogeneous subclones that exhibit dissimilar morphologies and self-renewal and proliferative capacities. Our results suggest that single cell-derived subclones from a patient can produce phenotypically heterogeneous self-renewing progenies in both in vitro and in vivo settings.

The overall treatment outcome of malignant brain tumors remains unsatisfactory even though advanced multimodal treatments including surgery, chemotherapy, and radiotherapy have been available for decades. The median survival of patients with glioblastoma multiforme (GBM), the most aggressive malignant brain tumor, is typically less than 2 years^1^. GBM, as its name suggests, is composed of a pathologically heterogeneous mixture of cells that exhibit varying degrees of cellular and nuclear polymorphism^2^. Although this heterogeneity is generally discussed in terms of pathological structures, examining the dynamic heterogeneity at the cellular level is fundamental to understanding the origins of the cells, potential therapeutic targets, and the source of tumor recurrences^3^. Therefore, functionally analyzing the individual types of the heterogeneous cells and determining their role in tumor pathogenesis are critical.

The cancer stem cell (CSC) hypothesis, which was first described in studies on leukemia, has attracted considerable attention in other cancer fields, including the one devoted to brain tumor^4^. Certain tumor cells exhibit stem cell-like characteristics and initiate tumors in animal models; thus, these cells are referred to as cancer stem-like cells or cancer-initiating cells^3^. Regardless of the nomenclatures used, the CSCs (as the cells are commonly termed) that are isolated directly from patients' tumors are considered to serve as valuable tools that can enhance our understanding of tumorigenesis, therapeutic resistances, and the functional heterogeneity of cancers in vitro and in vivo^5^.

In this study, we established 4 subclones from a glioblastoma patient and demonstrated clear evidence that a brain tumor contains heterogeneous subclones that exhibit dissimilar morphologies, self-renewal, proliferative capacities and therapeutic sensitivities.

## Results

### Growth-pattern differences in vitro

**Figure 1:**
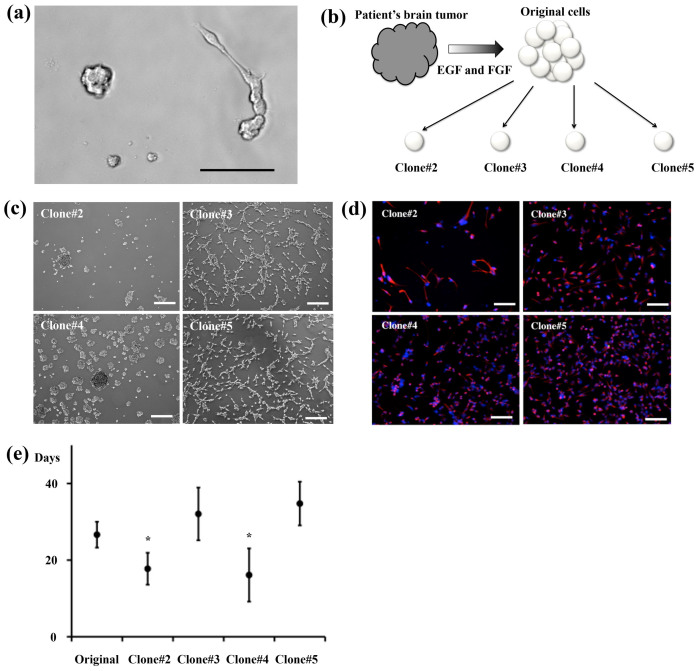
Various clones grown in vitro. (a) Distinct cellular morphologies observed after plating dissociated tumor cells in culture dishes. (b) Procedures used in establishing the 4 clones. Immediately after tumor spheres were formed from the patient's tissue, mechanically dissociated single cells were plated in small culture dishes. (c) The clones #2 and #4 formed sphere-like aggregates, whereas #3 and #5 attached to the uncoated culture dishes. (d) The 4 clones expressed the stem-cell marker nestin (red, nestin: blue, DAPI). (e) Cell-doubling time. Scale bars = 100 μm.

In laboratory settings, glioma stem cells (GSCs) can be isolated by adding EGF and FGF to suspension cultures in the absence of serum, which is the so-called sphere-forming method, or by using adherent culture systems in which specific materials are coated on culture dishes^4^,^6^. When either the sphere-forming system or the adherent system is used, some of the cancer cells can be propagated but other cells are lost because of cell death/apoptosis or differentiation. When we placed dissociated glioma tissues on uncoated culture dishes, some cells formed sphere-like aggregates, whereas other cells grew out and extended cellular processes (Fig. 1a). Although CD133 is the first-reported CSC marker of leukemia, glioma, and certain solid cancers, CD133-negative cells also possess CSC properties^4^,^7^. To study glioma heterogeneity, we established tumor-initiating clones from a single cell without fluorescence-activated cell sorting (FACS). After a few passages of the glioma tissue obtained from a 64-year-old wild-type IDH1 GBM patient, cells were mechanically dissociated into single cells and then placed individually in uncoated 96-well plates and cultured in the presence of EGF and FGF (Fig. 1b). After serial passages, 2 of the 4 clones were observed to grow as spheres, whereas the other 2 clones were found to adhere to the culture dishes (Fig. 1c). These clones homogenously expressed the neural/glioma stem-cell markers nestin (Fig. 1d), Sox2 (Supplementary Fig. 1a), and Musashi-1 (Supplementary Fig. 1b)^6^,^8^. The 2 sphere-forming clones, #2 and #4, displayed higher proliferative capacities than the original cells (i.e., the cells before dissociation into single cells), whereas the 2 adherent clones, #3 and #5, exhibited lower proliferative capacities than the sphere-forming clones (Fig. 1e). These data suggested that the patient's tumor tissue contained distinct proliferative clones, which were also morphologically dissimilar in vitro.

### Differences in tumorigenesis in an animal model

**Figure 2:**
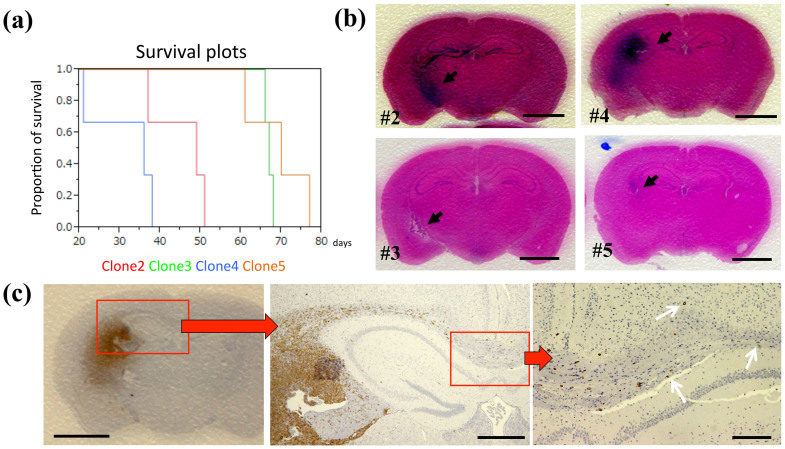
Dissimilar tumorigenesis in the in vivo animal model. (a) Kaplan-Meier survival plots; 3 mice were used for cells of each clone. (b) Representative brain tumors of NOD-SCID mice harboring xenografts of the clones #2 (upper left), #3 (lower left), #4 (upper right) and #5 (lower right); H.E. staining, arrows: tumor, scale bar = 5 mm. (c) A representative xenograft of the clone #4; nestin-positive cells infiltrated the contralateral hemisphere (arrows). Scale bars = 5 mm (left), 1 mm (middle), 50 μm (right).

Next, we evaluated the tumor-forming abilities of the clones by using an in vivo animal model. The cells of the clones #2 and #4 exhibited more extensive and well-defined tumor masses than did the cells of #3 and #5, and the sphere-forming clones induced higher mortality than did the other clones (Fig. 2a and 2b). The nestin-expressing GSCs were highly infiltrative: from the injection site, these cells invaded the contralateral hemispheres through the corpus callosum, thus reflecting the infiltrating behavior of GBM (Fig. 2c). These results demonstrate that the patient's tumor contained distinct infiltrative and growing cells. In clinical settings, such extensive infiltration of tumor cells into the surrounding healthy brain tissue prevents the removal of all tumor cells, and in animal models, these remaining GSCs offer enhanced resistance to irradiation and chemotherapy^9^. Our data may support the explanation that GSCs are left behind even after extensive resection and perhaps also after hemispherectomy^10^.

### Differences in cell-surface markers revealed through FACS analysis

**Figure 3:**
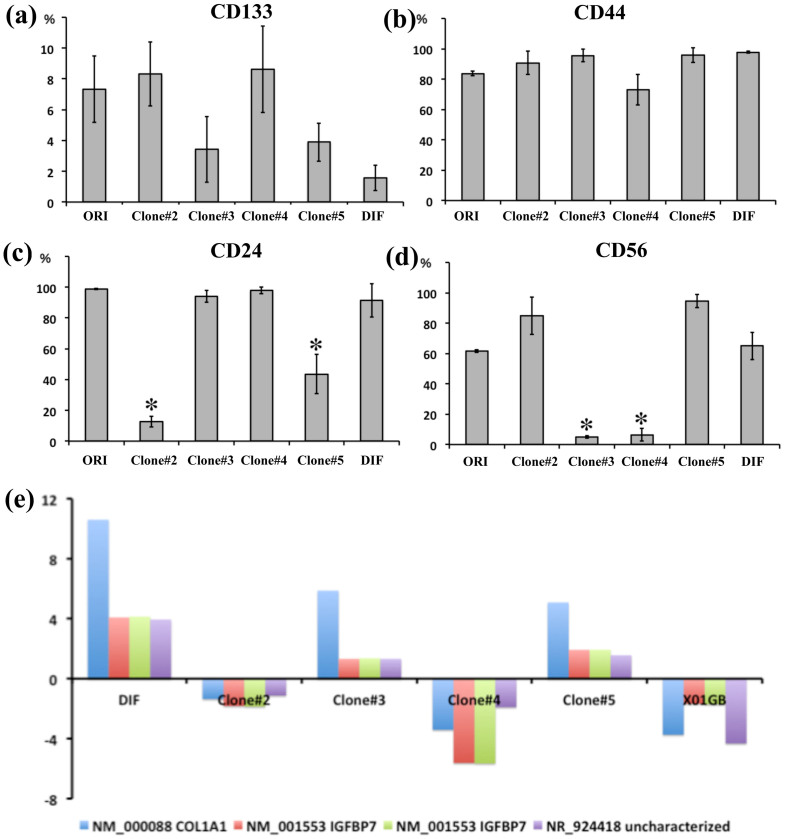
Analysis of cell-surface markers and cDNA arrays. Flow-cytometry data were collected in at least triplicate and at distinct culture periods in order to avoid ongoing culture selection. ORI: original cells not sorted into single cells; DIF: differentiated cells obtained using 10% serum. (a) CD133 expression (no statistically significant difference). (b) CD44 expression. (c) CD24 expression (*P < 0.01). (d) CD56 expression (*P < 0.01). (e) Analysis of cDNA arrays. Fold-changes in gene expression were calculated as the ratio of the signal values of the original cells to the value of each of the clonal cells after duplication.

Both organ-specific stem cells and CSCs give rise to phenotypically heterogeneous cells that exhibit varying degrees of differentiation and can be identified using specific markers. Although the prospective isolation of CSC populations from a tumor bulk is central to the CSC hypothesis, no single cell marker is adequate for identifying all CSCs. Flow cytometry can be used for distinguishing glial and neural downstream lineages of cells and for determining the degree of their differentiation by examining the expression of single surface markers or combinations of multiple surface markers^11^. To characterize the 4 clones, we investigated the expression of cell-surface markers by performing FACS analysis. We analyzed the following surface markers: CD133, as a marker of CSCs including GSCs^4^; CD44, for astroglial cells and CSCs; CD24, for neural stem cells and CSCs^12^; CD56, for neuronal cells^13^; and CD54 and CD166 as cell-surface proteins reported to be expressed in gliomas and cancers^14^,^15^,^16^. Furthermore, we analyzed the expression of EGF receptor (EGFR), which is expressed in immature astrocytes, is critical for astrocyte development, and is frequently overexpressed in high-grade glioma cells^17^. We also analyzed the expression of CXCR4 (a chemokine receptor), which is critically important for tumor invasion^18^. More CD133 was expressed in the clones #2 and #4 than in #3 and #5, although this difference was not statistically significant; nevertheless, this observation suggests that the clones expressing high levels of CD133 tended to exhibit high proliferative and invasive capacities (Fig. 3a). All clones expressed high levels of CD44, whereas the expression patterns of CD24 and CD56 showed statistically significant differences (Figs. 3b–d). However, the expression of CD24 and CD56 did not affect the morphology, proliferative capacities, and the tumorigenesis of the clones. The expression of the other tested markers varied, and we failed to identify any clear relationship between the markers and the phenotype of the clones in which they were expressed (Supplementary Fig. 2). These results suggested that after being cultured in vitro, the tumor tissue obtained from the patient exhibited at least 4 patterns of surface-marker expression: CD133high/CD24low/CD56high, CD133low/CD24high/CD56high, CD133high/CD24high/CD56low, and CD133low/CD24low/CD56high.

### IGFBP7 and collagen1A1 expression levels match clone characteristics

Next, we compared the gene-expression profiles of the clones by using cDNA microarrays. Data were analyzed using the Affymetrix GeneChip operating software, and fold-changes in gene expression were calculated as the ratio of the signal values of the bulk-sphere cells (original cells before cloning) to the values of each clone of cells. Only gene-expression changes with 2-fold significance were considered. The X01GB cell line, a stable and highly invasive GSC line, was used as the control GSC line^6^,^8^. Out of 58,721 probes, the cells of the clones #3 and #5 expressed collagen 1A1 (COL1A1), insulin-like growth factor binding protein 7 (IGFBP7), and the uncharacterized LOC389332 at higher levels than did the sphere-forming clones and resembled the cells that had differentiated under serum treatment (Fig. 1c, Fig. 2a, Fig. 3e, all 2-fold significant genes shown in Supplementary Table). According to the data of the Cancer Genome Atlas Research Network, both of these proteins are widely expressed in GBM patients (COL1A1, 95%; IGFBP7, up to 95%)^19^. Collagens are classified into 3 main classes and they play numerous roles in oncogenesis^20^. IGFBP7 has been implicated in cell proliferation and apoptosis, and the downregulation of IGFBP7 is associated with a poor clinical outcome in certain solid cancers^21^. Our result showed that #2 and #4, the clones in which COL1A1 and IGFBP7 were downregulated, induced high mortality in mice (Fig. 2a). These data suggest that COL1A1 and IGFBP7 may play critical roles in the development and tissue heterogeneity of gliomas. Since our four clones are insufficient number for analyzing p- or q-values, further evaluations of single cell derived clones may support our hypothesis.

### Distinct drug sensitivities in the 4 clones

**Figure 4:**
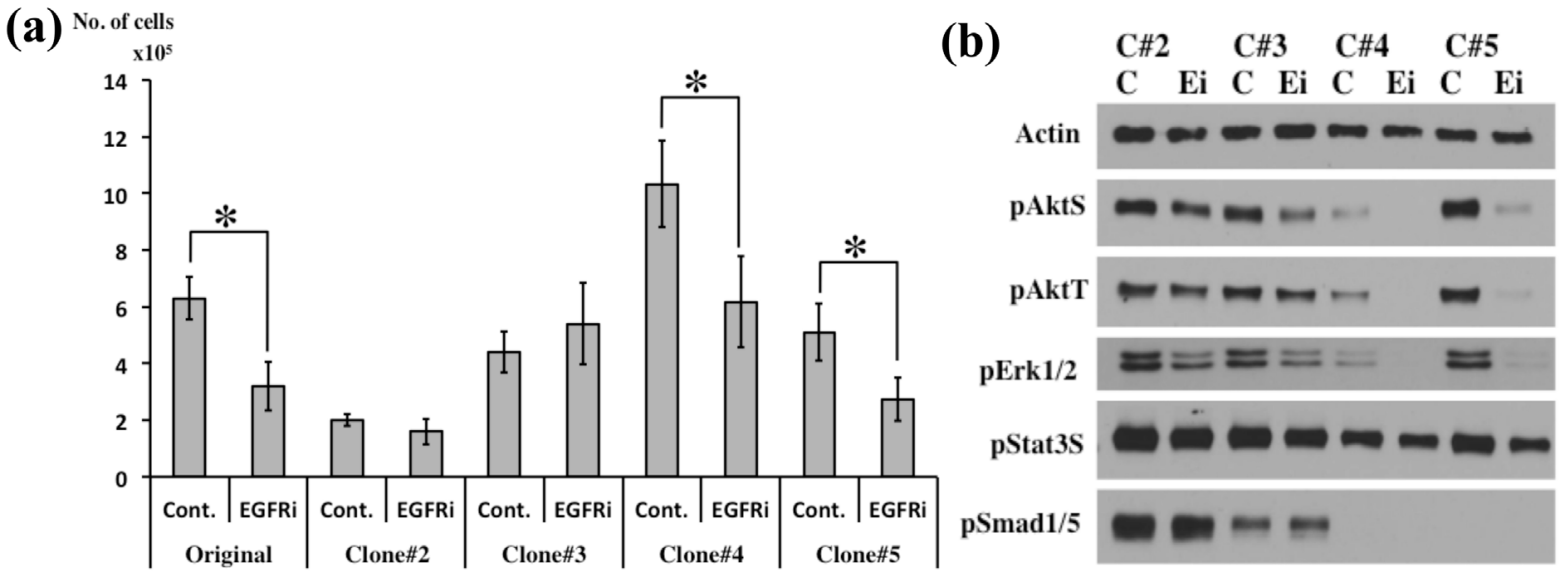
Inhibition of EGF-EGFR/PI3K/Akt and/or MAPK-Erk1/2 pathways by an EGFR inhibitor. (a) Cell numbers determined after treatment with the EGFR inhibitor gefitinib (1 μM); cells were counted after one week. *P < 0.01. Cont: control; EGFRi: EGFR inhibitor. (b) Phosphorylation of Akt, Stat3, Erk1/2, and Smad1/5 in cells incubated with 1 μM gefitinib for 1 h. C: control, Ei: EGFR inhibitor. Full-length blots are shown in Supplementary Fig. 4.

The capacity of CSCs to proliferate and differentiate can be modulated by both endogenous and environmental factors. Previously, we reported that the EGF-EGFR pathway plays a critical role in the proliferation of CD133-positive GSCs and that the PI3K-Akt and MAPK-Erk1/2 pathways are essential for the stem cell-like properties of GSCs^6^. In accordance with these findings, treatment with an EGFR inhibitor (gefitinib) decreased the number of cells of the clones #4 and #5, but the cells of #2 and #3 were unaffected by this inhibitor (Fig. 4a). Furthermore, after gefitinib treatment, the phosphorylation of Akt and Erk1/2 was either eliminated or diminished in the cells of the clones #4 and #5, whereas the phosphorylation of these proteins was retained in the cells of #2 and #3 (Fig. 4b, full lengths of blots shown in Supplementary Fig. 3). These data suggest that the tumor obtained from the patient contained both gefitinib-sensitive and gefitinib-resistant cells that displayed dissimilar therapeutic responses. Malignant gliomas often exhibit p53 mutation and/or EGFR amplification, and the tumor suppressor PTEN (phosphatase and tensin homolog) is frequently deleted in GBM, leading to downstream activation of the Akt pathway^17^,^19^. However, DNA sequencing revealed no mutation or amplification of p53, PTEN, or EGFR in the 4 clones studied here. Therefore, multiple mechanisms and/or additional mutational events may enable each of these 4 single-cell clones to survive.

## Discussion

The development of technical abilities to grow cells dissociated from tissues in plastic dishes and the ability to identify these cells have enhanced the understanding of both normal and cancer biology. The notion of CSCs is highly novel and could be of considerable significance in the cancer field, and the idea of cancer heterogeneity has recently emerged from the field of stem cell biology. Because cancer can be considered a disease of unregulated self-renewal and differentiation, understanding cancer heterogeneity is fundamental to understanding cancer-cell proliferation. As indicated by the word “multiforme”, GBMs display a highly heterogeneous composition of cells, and GBMs exhibit phenotypic heterogeneity because they are composed of cells that express markers of both undifferentiated and differentiated cells^2^. However, the degree of differentiation and the types of cells produced differ from tumor to tumor, possibly reflecting a fundamental dissimilarity in the progenitor cells of distinct tumors. In this study, we obtained clear evidence that a brain tumor contained heterogeneous tumor cells that exhibited dissimilar morphologies and self-renewal and proliferative capacities in both in vitro and in vivo settings. Recently, Patel et al. demonstrated intratumoral heterogeneity in primary GBMs by using the single-cell RNA-sequencing method^22^; the researchers investigated spheroids and differentiated clusters that resemble our 2 morphologically distinct types of cells, but they did not use in vivo animal models. The cells of our clones #3 and #5 resembled differentiated cells and grew as monolayers, but they induced tumorigenesis in the mouse brain; thus, clusters of cells that resemble differentiated cells might not represent true differentiated GSCs. The 4 clones examined in this study exhibited dissimilar drug sensitivities and they might use distinct proliferation pathways. Patel et al. also reported that mosaic RTK amplification contributed to drug resistances, and the lack of EGFR in several clones suggested that potential alternative pathways are used for proliferation signaling^22^. Although we have not conclusively identified the mechanism that can explain such differences, the findings suggest that such mechanisms include distinct RTK signaling pathways that are responsible for GBM proliferation. Further evaluating the underlying metabolic events in heterogeneous cancer cells and assessing the biological features of each distinct type of cancer cells will yield valuable information on the in situ behavior of cancers and help identify optimal cell-specific therapies in the future.

## Methods

### Cell cultures

Tumor cells were derived from a 64-year-old GBM patient. Prior informed consent was obtained from the donor and the donor's family. Tumor-sphere cultures were prepared as described previously, with some modifications, in DMEM-F12 (GIBCO-Invitrogen, La Jolla, CA) containing penicillin G, streptomycin sulfate, B-27 (GIBCO-Invitrogen), recombinant human epidermal growth factor (EGF) (20 ng/mL; R&D Systems, Minneapolis, MN), and recombinant human fibroblast growth factor 2 (FGF2) (20 ng/mL; R&D Systems)^4^,^6^. Cells were cultured in HERAcell incubators (Thermo Electronic Corporation, Asheville, NC) at 37°C, ≥95% relative humidity, and 5% CO_2_ under 20% oxygen conditions. We count cell numbers at each passage period with a hemacytometer in trypan blue staining as usual manner^6^.

### In vivo experiments

Tumorigenicity was determined by injecting tumor cells orthotopically into non-obese diabetic-severe combined immunodeficiency (NOD-SCID) mice (SLC, Shizuoka, Japan). Cells were injected into the brain of ketamine-anesthetized NOD-SCID mice; 2 μL of a cell suspension (1 × 10^8^ cells/mL) in proliferation medium were delivered into the right striatum (1 μL/min) by using a stereotactic instrument (SR-60, Narishige, Tokyo, Japan) and a Hamilton syringe. The injection coordinates were 3 mm to the right of the midline and 3 mm anterior to the lambda, at a depth of 3 mm. We injected 3 mice each with the cells of each clone, and all of the mice were monitored daily for signs of morbidity such as weight loss, seizures, posturing, and nasal and/or periorbital hemorrhage; the mice were sacrificed at the first sign of morbidity, and the collected brains were examined histologically for the presence of tumor.

### Immunohistochemical analysis of brain tissues

Tumor samples were fixed in 4% paraformaldehyde, embedded in paraffin, and cut into 3-μm sections. For hematoxylin-eosin staining, sections were first stained with Mayer's hematoxylin (1 min) and then counterstained with alcoholic eosin. For immunohistochemical studies, deparaffinized sections were washed in Tris-buffered saline (TBS) and endogenous peroxidase was neutralized using 3% H_2_O_2_ in methanol (15 min) after 15-min antigen retrieval in citrate buffer in a microwave at 500 W. Sections were blocked with 1% bovine serum albumin in TBS and then treated overnight at 4°C with the following primary antibodies: anti-human nestin (mouse monoclonal antibody (mAb), 5 μg/mL; R&D Systems) for glioma CSCs. After treatment with biotinylated secondary antibodies and horseradish peroxidase (HRP)-linked streptavidin (LSAB2 kit, DAKO), color reactions were performed using the peroxidase substrate 3,3′-diaminobenzidine (DAB, DAKO). All sections were counter-stained with Mayer's hematoxylin.

### FACS analysis

Cells were evaluated on a Coulter EPICS cytometer (Beckman Coulter, Fullerton, CA). To characterize glioma CSCs, each sample was labeled with phycoerythrin (PE)-conjugated anti-human CD24, CD44, CD54, CD56, CD166, CXCR4 and EGFR antibodies (BD Biosciences, San Jose, CA), PE-conjugated CD133/1 (AC133) (Miltenyi Biotec, Auburn, CA) according to the manufacturer's recommendation. All data used unstained control and used Cellquest Pro software/FlowJo for data acquisition and analysis including adequate fluorescence labeling, spectrum, compensation and calibration at flow cytometry facilities in University of Pittsburgh and/or University of Virginia. All experiments were performed in triplicate.

### Western blotting

Western blot analyses were performed as described previously^6^. Antibodies against the following molecules were used: actin and phospho-STAT3 (P-Ser727) (Santa Cruz Biotechnology, Santa Cruz, CA), and phospho-Akt (P-Ser473), phospho-Akt (P-Thr308), phospho-ERK1/2 (P-Thr202/Tyr204), and phospho-Smad1/5 (P-Ser463/465) (Cell Signaling Technology, MA). Briefly, tumor cells were lysed in a buffer consisting of 20 mM Tris-HCl, pH 7.4, 150 mM NaCl, 1 mM EGTA, 1% Triton X-100, 2.5 mM sodium pyrophosphate, 1 mM β-glycerol phosphate, 1 mM Na3VO4, 1 μg/mL leupeptin, and 1 mM phenylmethylsulfonyl fluoride. After brief sonication, lysates were clarified by centrifugation at 12,000 × *g* for 10 min at 4°C, and the protein content in the supernatant was measured according to the Bradford method. Aliquots (40 μg of protein per lane) of total protein were separated using SDS-polyacrylamide gel electrophoresis (7.5% gels) and blotted onto nitrocellulose transfer membranes (0.2 μm; Amersham Biosciences, Buckinghamshire, UK). Each membrane was blocked with 5% non-fat dry milk in TBS-T (20 mM Tris-HCl, pH 7.6, 137 mM NaCl, and 0.01% Tween-20) for 1 h at room temperature, and then incubated with the appropriate primary antibodies overnight at 4°C. After extensive washing with TBS-T, each membrane was further incubated (1 h, room temperature) with HRP-conjugated anti-rabbit, anti-mouse, or anti-goat secondary antibodies diluted (1:1,000) in TBS-T containing 5% non-fat dry milk. Immunoreactive bands were detected using an enhanced chemiluminescence reagent (Amersham Biosciences), according to the manufacturer's protocol.

### Microarray procedures and data analysis

The preparation of cRNA, hybridization, and scanning of the microarrays were performed according to the manufacturer's protocol (Affymetrix, Santa Clara, CA); analysis was on both a spectrophotometer and an Agilent 2100 Bioanalyzer (Agilent Technologies, Palo Alto, CA). Total RNA was extracted from each sample by using TRIZOL reagent (Invitrogen); this was followed by passage through an RNeasy spin column (Qiagen, Valencia, CA) and amplification with RiboAmp RNA Kits (Arcturus Engineering, Mountain View, CA) according to the manufacturer's protocol. Amplified RNA (7.5 μg) was labeled with Cy5-dUTP (experimental RNA) or Cy3-dUTP (Stratagene, La Jolla, CA) by using Superscript II reverse transcriptase (Invitrogen). The labeled cRNA was hybridized to the Affymetrix Human Genome U133 Plus 2.0 Genechip, with the use of 60-rpm rotation for 16 h at 45°C. After hybridization, the microarrays were washed in a buffer containing biotinylated anti-streptavidin antibodies (Vector Laboratories, Burlingame, CA) and stained (10 min at 25°C) with streptavidin-PE (final concentration 10 μg/mL; Molecular Probes, OR). Subsequently, the microarrays were washed, restained with streptavidin-PE, and washed again before measuring fluorescence at 570 nm in the Affymetrix GeneChip Scanner 3000. All of the microarrays were examined for surface defects, grid placement, background intensity, housekeeping gene expression, and the 3′/5′ ratio of probe sets from genes of varying length (signal 3′/5′ ratio < 3). Initial data analysis was performed using Affymetrix Microarray Suite 5.0 to determine gene expression levels.

### Ethics Statement

Our study was approved by the Medical Review Boards of University of Pittsburgh, University of Virginia, and Gifu University School of Medicine. Our experimental procedures involving animals also followed the guidelines of the Animal Experimental Committee of Gifu University.

### Statistical analysis

Statistical analysis was performed using Student's *t* tests; *P* < 0.05 was considered statistically significant.

## Author Contributions

A.S. wrote the manuscript. A.S. designed and performed experiments and A.H. performed histological studies. S.Y. assisted with experiments. S.Y., T.K., T.I. and D.M.P. helped with the evaluation of the project. All authors reviewed the manuscript.

## Supplementary Material

Supplementary Information Supplementary Figures1-3 and Table
