# Supplementary material for: The Evidence of Glioblastoma Heterogeneity
Source: Sci Rep. 2015 Jan 27;5:7979. doi: 10.1038/srep07979 (PMC4306917; doi:10.1038/srep07979)
Supplement: Supplementary Information — Supplementary Figures1-3 and Table [file srep07979-s1.pdf]

Title:

The Evidence of Glioblastoma Heterogeneity

Authors:

Akio Soeda, Akira Hara, Takahiro Kunisada, Shin-ichi Yoshimura, Toru Iwama, Deric M. Park

Supplementary Figure1a.   The 4 clones expressed the stem cell marker Sox2 (red). Scale bars = 100 μm.

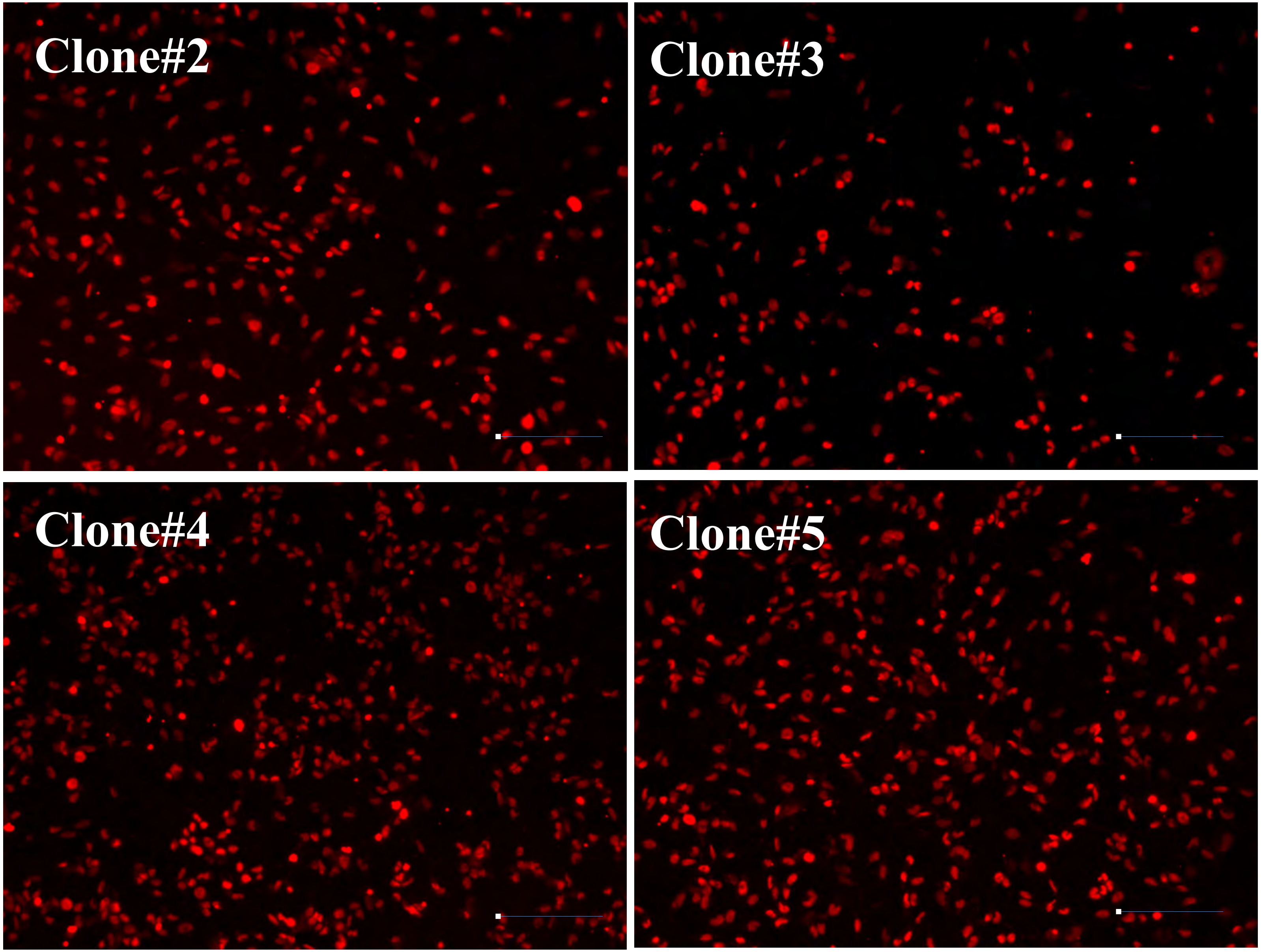

Supplementary Figure1b. The 4 clones expressed the stem cell marker Musashi (green). Scale bars = 100 µm.

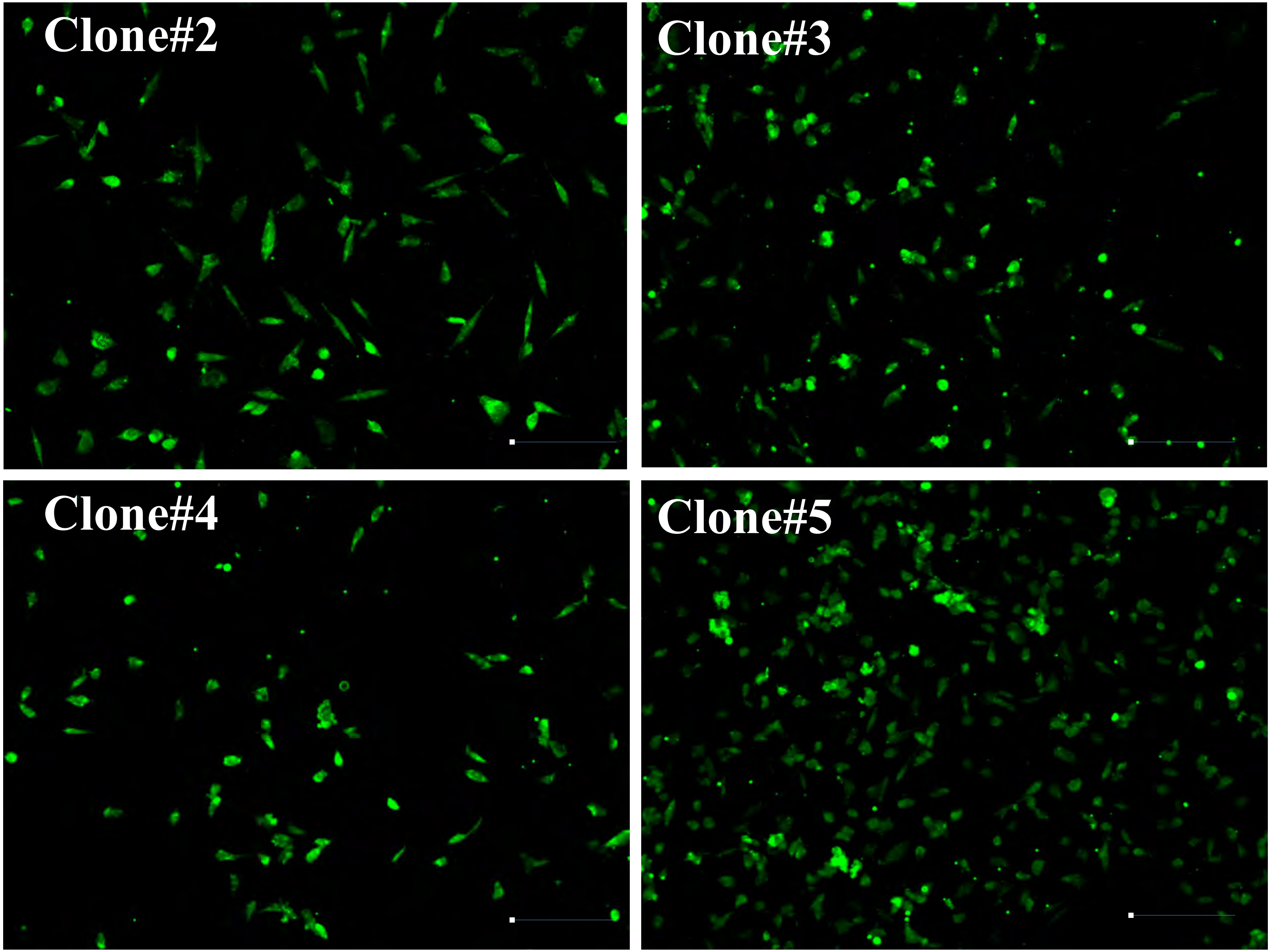

Supplementary Figure2. FACS analysis of EGFR, CXCR4, CD54 and CD166.

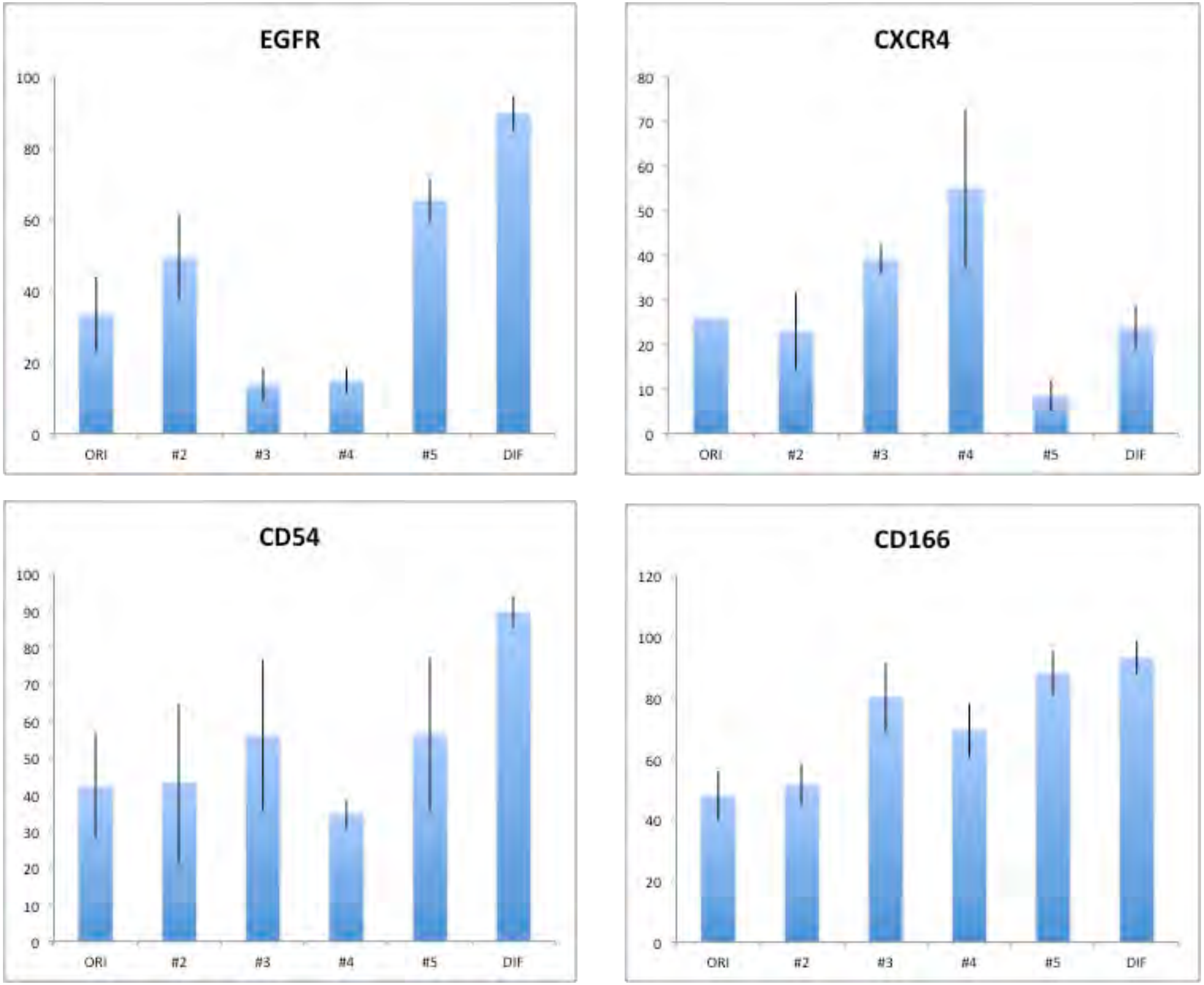

Supplementary Figure 3. Full length of blots seen in Figure 3b.

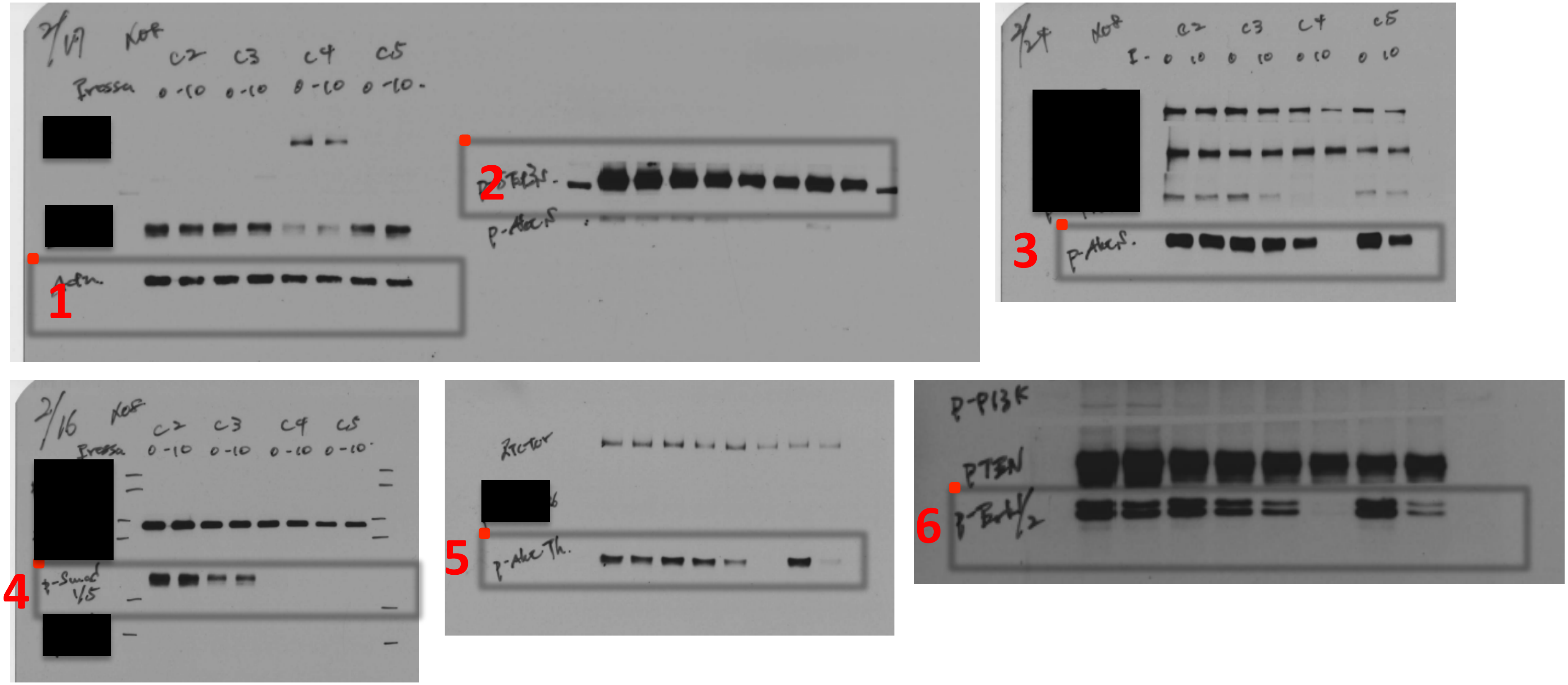

1)Actin, 2)p-Stat3S, 3)p-AktS, 4)p-Smad1/5, 5)p-AktT, 6)p-Erk1/2

Supplementary Table1. Fold-changes in the values for genes were calculated as the ratio of the signal values of the bulk-sphere cells (original cells before cloning) to the values of each clone of cells. Only gene expression changes with 2-fold significance are shown.

GeneSymbol	GenbankAccession	DIF	Clone#2	Clone#3	Clone#4	Clone#5	X01GB
AKR1B10	NM_020299	1.84	-1.11	-1.73	-1.73	-1.02	4.03
AKR1B10	NM_020299	1.87	-1.38	-1.84	-2.10	-1.19	3.95
AKR1B15	NM_001080538	1.81	-1.08	-1.68	-2.19	-1.15	3.98
ANGPTL1	NM_004673	-5.66	2.29	2.11	1.30	2.24	-6.41
ANGPTL1	NM_004673	-3.66	2.47	2.09	1.18	2.39	-6.05
ANGPTL4	NM_139314	5.15	-2.00	-1.73	-2.70	-3.37	-5.05
ANGPTL4	NM_139314	5.27	-2.01	-1.95	-2.89	-3.16	-4.24
ANKRD20A5P	BC022023	-3.48	-1.74	-1.93	-2.00	-1.30	-3.20
ANKRD20A9P	NR_027995	-2.76	-1.13	-1.18	-1.72	-1.38	-4.47
ATXN7L1	BC003517	-5.05	-2.82	-2.71	-2.51	-1.21	-1.08
BST2	NM_004335	-3.57	-1.22	-1.55	-1.51	-2.12	1.05
C21orf122	NR_027292	5.10	2.83	3.76	2.75	3.82	2.56
C21orf37	NR_037585	-5.70	-2.21	-2.69	-1.59	-1.87	-2.51
C3	NM_000064	1.56	-1.34	-3.43	-3.36	-2.64	4.30
CABLES1	NM_138375	1.41	-1.04	-1.23	-1.20	-1.23	-5.79
CCDC165	NM_015210	3.83	1.49	1.33	1.60	1.81	1.03
CCND1	NM_053056	2.89	1.22	1.02	1.31	1.35	1.01
CCND1	NM_053056	2.90	1.18	1.01	1.30	1.49	1.02
CCND1	NM_053056	2.95	1.31	1.07	1.44	1.51	1.07
COL1A1	NM_000088	10.60	-1.35	5.86	-3.40	5.08	-3.73
COL8A2	NM_005202	1.06	-2.72	-1.98	-4.40	-1.38	-3.24
COX8C	NM_182971	-4.75	-2.00	-1.65	-1.94	-1.25	-3.01
CRYAB	NM_001885	3.41	-2.39	-2.71	-2.58	-2.50	-2.31
CRYAB	NM_001885	3.42	-2.40	-2.58	-2.58	-2.46	-2.29
CTGF	NM_001901	6.09	2.42	3.18	1.72	1.80	-4.15
CTGF	NM_001901	6.16	2.53	3.29	1.75	1.72	-4.71

Supplementary Table1. (Continued)

DPF3	AK024141	−2.44	1.19	3.12	1.40	1.13	2.25
DPF3	AK024141	−1.36	1.07	3.07	1.61	1.09	2.66
EBI3	NM_005755	1.84	−1.10	−1.23	−1.15	−2.03	−2.68
EDIL3	NM_005711	1.33	−4.32	−2.54	−5.20	−4.63	−3.19
EFNA1	NM_004428	−3.30	−1.06	−1.25	−1.20	−2.13	−3.00
ESR2	NM_001437	−3.86	1.59	1.08	1.38	3.33	1.41
ESR2	NM_001437	−3.82	1.29	1.03	1.34	3.33	1.30
ESR2	NM_001437	−3.79	1.67	1.12	1.54	3.47	1.57
ESR2	NM_001437	−3.57	2.04	1.66	2.08	3.85	1.98
ESR2	NM_001437	−3.56	1.86	1.22	1.73	3.69	1.80
ESR2	NM_001437	−3.44	1.93	1.51	1.87	3.73	1.77
ESR2	NM_001437	−2.52	2.20	1.34	2.00	3.89	1.95
ESR2	NM_001437	−2.10	1.80	1.21	1.60	3.56	1.76
FAM134B	NM_001034850	−3.83	−1.79	−1.55	−1.05	−1.99	−2.08
FBRSL1	BC013284	3.22	1.32	1.70	1.37	1.97	1.86
FHL2	NM_001039492	5.79	1.21	1.04	2.39	−1.27	−4.17
FLJ45684	AK127589	5.35	2.49	2.40	1.84	2.47	2.71
FZR1	NM_016263	3.15	1.19	1.56	1.06	1.54	1.52
GBP2	NM_004120	−1.04	−3.24	−2.47	−2.30	−2.99	−2.44
GRPR	NM_005314	5.72	2.32	2.49	1.83	3.64	4.83
HBEGF	NM_001945	3.83	1.60	1.40	1.81	1.39	4.38
HDAC9	NM_058176	4.41	1.88	1.98	2.04	3.12	2.31
HES7	NM_001165967	−4.00	−1.29	−1.77	−1.59	−1.62	−3.64
HSPB2	NM_001541	1.22	−1.49	−1.61	−1.47	−1.95	−1.59
ID1	NM_002165	2.56	−2.02	−2.06	−1.96	−4.68	−2.01
ID1	NM_002165	2.60	−2.03	−2.16	−1.99	−4.64	−2.08
IGFBP7	NM_001553	4.08	−1.81	1.32	−5.61	1.92	−1.66
IGFBP7	NM_001553	4.14	−1.85	1.36	−5.64	1.93	−1.70
KYNU	NM_001032998	−1.99	−4.10	−4.51	−3.19	−4.86	−7.04
LOC100131262	AK092421	4.72	1.84	2.84	1.65	2.26	2.93
LOC339929	NR_036497	4.75	2.61	3.35	2.74	3.23	2.07
LOC389332	NR_024418	3.94	−1.12	1.32	−1.89	1.56	−4.31
MSMP	NM_001044264	−4.31	1.50	5.61	4.13	5.30	9.01
NDNF	NM_024574	1.58	−1.13	−1.80	−1.75	−1.11	−2.86

Supplementary Table1. (Continued)

NFIA	NM_001134673	−2.07	1.31	1.41	1.03	1.58	5.40
NPAS1	NM_002517	1.05	−1.55	−1.47	−1.23	−1.35	−1.81
PACS2	NM_001100913	2.61	1.16	1.55	1.20	1.10	1.44
PRINS	NR_023388	−1.60	2.00	2.09	1.39	1.88	2.29
S1PR5	NM_030760	−2.67	−1.14	−1.06	−1.62	−1.10	−5.62
SEMA3F	NM_004186	2.81	−1.44	−1.44	−1.15	−1.71	−1.41
SERPINB8	NM_001031848	4.09	2.18	2.83	2.22	2.35	1.00
SEZ6L2	NM_201575	1.42	−1.61	2.95	−2.70	−2.74	2.06
SLC2A3	NM_006931	−2.73	−1.23	−1.14	−1.07	−1.06	−3.36
SLC37A2	NM_198277	7.28	2.61	2.27	1.87	4.38	2.77
SLC6A15	NM_018057	−2.32	−1.12	−4.88	−1.13	−4.90	−1.07
SLCO4A1	NM_016354	−2.32	1.83	1.27	2.15	2.47	1.35
SLN	NM_003063	2.12	−2.73	−3.94	−4.06	−2.63	−6.85
SMAD9	NM_005905	1.49	−1.36	−1.59	−1.79	−2.50	−1.32
SMAD9	NM_001127217	1.52	−1.55	−1.72	−1.99	−3.68	−1.30
SMAD9	NM_001127217	1.76	−1.41	−1.66	−2.28	−2.92	−1.17
SOHLH2	NM_017826	−4.21	−1.01	−2.54	−2.71	−1.40	−2.05
SP2	NM_003110	2.25	1.11	1.10	1.05	1.03	1.54
ST6GALNAC2	NM_006456	2.48	−1.62	−2.68	−1.02	−1.80	−1.99
TNNT1	NM_003283	1.59	−2.06	−1.83	−2.33	−3.66	−3.40
TNNT1	NM_003283	1.66	−2.15	−1.87	−2.29	−3.60	−3.36
TPMT	NM_000367	2.69	1.19	1.11	1.08	1.47	1.82
